# Development and Validation of an HPLC Method for Simultaneous Quantification of Clopidogrel Bisulfate, Its Carboxylic Acid Metabolite, and Atorvastatin in Human Plasma: Application to a Pharmacokinetic Study

**DOI:** 10.1155/2015/892470

**Published:** 2015-12-29

**Authors:** Octavian Croitoru, Adela-Maria Spiridon, Ionela Belu, Adina Turcu-Ştiolică, Johny Neamţu

**Affiliations:** ^1^Faculty of Pharmacy, Department I of Pharmacy, University of Medicine and Pharmacy, Petru Rares Street, 200349 Craiova, Romania; ^2^Faculty of Pharmacy, Doctoral School, University of Medicine and Pharmacy, Petru Rares Street, 200349 Craiova, Romania; ^3^Faculty of Pharmacy, Department II of Pharmacy, University of Medicine and Pharmacy, Petru Rares Street, 200349 Craiova, Romania

## Abstract

A simple, sensitive, and specific reversed phase liquid chromatographic method was developed and validated for simultaneous quantification of clopidogrel, its carboxylic acid metabolite, and atorvastatin in human serum. Plasma samples were deproteinized with acetonitrile and ibuprofen was chosen as internal standard. Chromatographic separation was performed on an BDS Hypersil C_18_ column (250 × 4.6 mm; 5 *μ*m) via gradient elution with mobile phase consisting of 10 mM phosphoric acid (sodium) buffer solution (pH = 2.6 adjusted with 85% orthophosphoric acid) : acetonitrile : methanol with flow rate of 1 mL·min^−1^. Detection was achieved with PDA detector at 220 nm. The method was validated in terms of linearity, sensitivity, precision, accuracy, limit of quantification, and stability tests. Calibration curves of the analytes were found to be linear in the range of 0.008–2 *μ*g·mL^−1^ for clopidogrel, 0.01–4 *μ*g·mL^−1^ for its carboxylic acid metabolite, and 0.005–2.5 *μ*g·mL^−1^ for atorvastatin. The results of accuracy (as recovery) with ibuprofen as internal standard were in the range of 96–98% for clopidogrel, 94–98% for its carboxylic acid metabolite, and 90–99% for atorvastatin, respectively.

## 1. Introduction

Numerous international guidelines provide evidence based recommendations of coprescribing antiplatelets drugs and statins in secondary prevention of cardiovascular events in patients with atherothrombosis (acute coronary syndromes (ACS), cerebrovascular disease, and peripheral arterial disease (PAD)) [1, 2].

**Figure 1:**
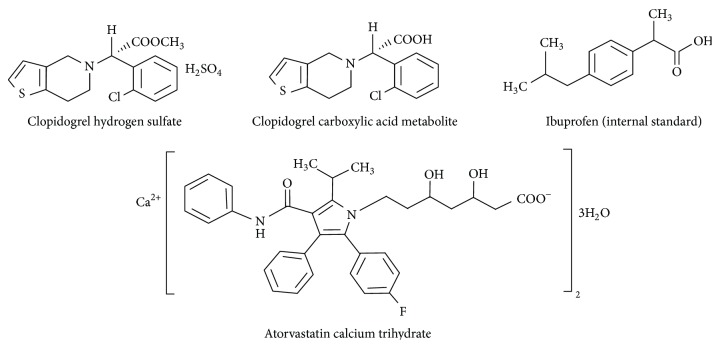
Chemical structure of clopidogrel hydrogen sulfate, its carboxylic acid metabolite, atorvastatin, and ibuprofen (internal standard).

Clopidogrel hydrogen sulfate,* methyl (2S)-(2-chlorophenyl)[6,7-dihydrothieno[3,2-c]pyridin-5(4H)-yl]acetate sulfate* (Figure 1), remains the oral thienopyridine agent most prescribed in the world with a loading dose of 300 or 600 mg and maintenance dose of 75 mg [2]. Clopidogrel is a prodrug converted about 15% in the liver by cytochrome P450 enzymes in a two-step process to the thiolic active metabolite [3] that irreversibly blocks the P2Y12 receptor by disulfide bonding. The first step involves cytochrome P450-dependent monooxygenation [4] to 2-oxo-clopidogrel and the second cytochrome P450-dependent oxidative opening [4] of the thiolactone ring to an intermediate sulfenic acid metabolite subsequently reduced to the active thiolic metabolite. A recent paper described PON-1-dependent hydrolysis of 2-oxo-clopidogrel leading to endometabolites [5]. The majority (85%) of the prodrug is hydrolyzed by esterases to an inactive carboxylic acid metabolite.

Statins are inhibitors of 3-hydroxyl-methylglutaryl coenzyme A (HMG-CoA) reductase and are recommended for NSTE-ACS patients as early as possible to reduce low-density lipoprotein cholesterol (LDL-c) levels of 2.6 mmol·L^−1^ (<100 mg·dL^−1^), the maximal benefit being achieved with high dose (80 mg atorvastatin) [1].

Atorvastatin, calcium (3R,5R)-*7-[2-(4-fluorophenyl)-5-(1-methylethyl)-3-phenyl-4-(phenylcarbamoyl)-1H-pyrrol-1-yl]-3,5-dihydroxyheptanoate trihydrate* (Figure 1), is widely used to reduce morbidity and mortality in patients with atherosclerosis and cardiovascular disease through anti-inflammatory, antioxidative, and antithrombotic effects. Lactonization of the acid form and hydrolysis of the lactone form to open acid form are catalyzed by esterases and uridine diphosphate (UDP) glucuronosyltransferase [6, 7]. The lactone forms of atorvastatin have no lipid-lowering effects, orthohydroxyl atorvastatin is the main active metabolite detected in plasma, and parahydroxyl atorvastatin has low plasma concentration [7]. Atorvastatin has low bioavailability of about 12% when administered orally due to presystemic clearance in intestine and metabolization in the liver involving cytochrome P450 oxidation [8]. Atorvastatin has been studied in clopidogrel trials because of its high affinity to the CYP3A4 isoenzyme, causing a possible loss of antiplatelet effect of clopidogrel. Despite some clinical trials suggesting a drug-drug interaction between clopidogrel and atorvastatin, there is no significant clinical evidence to stop their coadministration in patients at high risk of atherothrombotic events.

Most published reports describe the quantification of clopidogrel carboxylic acid metabolite in biological matrix using either LC-UV for human plasma [9, 10] and rat plasma [11], LC-MS [12, 13], or LC-MS/MS [14–20]. Because of its low plasma concentration and its insufficient stability, the thiolic metabolite has been detected in few reports by LC-MS/MS [21–24].

Various methods have been applied for quantification of atorvastatin in human plasma by LC-UV [25, 26], for simultaneous estimation of atorvastatin and its active metabolites in human plasma by LC-MS [27, 28] and alone [29]. Two reports describe simultaneous determination of atorvastatin, its orthohydroxyl and parahydroxyl metabolites, and amlodipine in human plasma by LC-MS/MS [30, 31] and one for atorvastatin, metformin, and glimepiride [32]. Two reports describe simultaneous determination of atorvastatin and rosuvastatin [33], atorvastatin, and simvastatin, respectively [34], in human serum.

The present paper describes a selective gradient chromatographic method for simultaneous determination of clopidogrel, its carboxylic acid metabolite, and atorvastatin in human plasma and* in vivo* application in three patients following oral administration of clopidogrel and atorvastatin during their maintaining therapy. The method was applied to investigate whether a potentially harmful pharmacokinetic interaction occurs between clopidogrel and atorvastatin in patients with antiplatelet and statin therapy.

## 2. Experimental

### 2.1. Materials and Reagents

Clopidogrel bisulfate, its carboxylic acid metabolite, atorvastatin calcium, and ibuprofen standards were obtained from Sigma Aldrich. HPLC grade acetonitrile, methanol, orthophosphoric acid, and water were obtained from Merck KGaA (Germany).

### 2.2. Instrumentation

Chromatographic analysis was carried out using a Thermo Finnigan chromatograph consisting of ternary solvent manager, a manual injector of 20 *μ*L loop, PDA detector, and a Thermo Finnigan Xcalibur software for data acquisition. The separation was achieved on an BDS Hypersil C_18_ analytical column (250 × 4.6 mm, 5 *μ*m particle size).

### 2.3. Preparation of Stock and Working Standard Solutions

The stock solutions of clopidogrel bisulfate, its carboxylic acid metabolite, atorvastatin, and ibuprofen (IS) were prepared at a concentration of 100 *μ*g·mL^−1^ in methanol and stored at 4°C. The working standard solutions for calibration curves have been prepared by serial dilutions with methanol at the concentrations of 50, 10, and 1 *μ*g·mL^−1^. Working standard solution of the IS (50 *μ*g·mL^−1^) was prepared in methanol. A 2 M hydrochloric acid solution was prepared in distilled water.

### 2.4. Preparation of Calibration Standards and Quality Control Samples

For calibration curves, human plasma (0.5 mL) was successfully spiked with clopidogrel bisulfate, its carboxylic acid metabolite, and atorvastatin working solution to final plasmatic concentrations of 0.008, 0.016, 0.032, 0.064, 0.128, 0.25, 0.5, 1, and 2 *μ*g·mL^−1^ for clopidogrel bisulfate, 0.015, 0.03, 0.06, 0.125, 0.5, and 4 *μ*g·mL^−1^ for its carboxylic acid metabolite, and 0.005, 0.01, 0.02, 0.04, 0.08, 0.16, 0.32, 0.64, 1.28, and 2.56 *μ*g·mL^−1^ for atorvastatin. To each calibration standard, IS (ibuprofen) was added in final plasmatic concentration of 2 *μ*g·mL^−1^ (20 *μ*L of working solution 50 *μ*g·mL^−1^). The quality control samples (QCs) used in method validation were spiked at the levels: 0.25, 0.5, 1, and 2 *μ*g·mL^−1^ *μ*g·mL^−1^ for clopidogrel bisulfate, 0.5, 1, 2, and 4 *μ*g·mL^−1^ for its carboxylic acid metabolite, and 0.2, 0.3, 1, and 2.5 *μ*g·mL^−1^ for atorvastatin.

### 2.5. Sample Preparation Procedure

All samples were identically treated as follows: to 0.5 mL plasma samples, 20 *μ*L ibuprofen standard solution (50 *μ*g·mL^−1^), 20 *μ*L working standard solutions of analytes solution, and 200 *μ*L of HCl solution (2 M) were added. Protein precipitation was obtained with 500 *μ*L of acetonitrile. The samples were shaken for 5 minutes and centrifuged for 10 minutes at 4500 rpm. Then, the supernatant was transferred into conical tubes and evaporated to dryness under gentle stream of nitrogen at ambient temperature. The residue was reconstituted in 500 *μ*L methanol and 20 *μ*L was injected into chromatographic column.

### 2.6. Chromatographic Conditions

The separation was achieved on an BDS Hypersil C_18_ analytical column (250 × 4.6 mm, 5 *μ*m particle size). The mobile phase consisted of A, 10 mM phosphoric acid (sodium) buffer solution (pH = 2.6 adjusted with 85% orthophosphoric acid); B, acetonitrile; and C, methanol. The gradient scheme is provided in Table 1. The total run time was 20 minutes, flow rate was 1 mL·min^−1^, and injection volume was 20 *μ*L. The UV detection was performed at 220 nm.

### 2.7. Method Validation

The method proposed was validated as described in ICH guidelines [35] in terms of linearity, limit of detection and quantification, accuracy, precision, and stability tests.

#### 2.7.1. Linearity

The linearity of the method was determined at eight concentration levels ranging from 0.008 to 2 *μ*g·mL^−1^ for clopidogrel bisulfate, 0.01 to 4 *μ*g·mL^−1^ for its carboxylic acid metabolite, and 0.005 to 2.5 *μ*g·mL^−1^ for atorvastatin. The calibration curves were constructed by plotting the peak areas ratio (area analytes/area IS) versus concentration of analytes. The slope, Y-intercept, and correlation coefficient were calculated.

#### 2.7.2. Limit of Detection (LOD) and the Lower Limit of Quantification (LLOQ)

The limit of detection (LOD) was estimated using signal-to-noise ratio of 3 : 1 and the lower limit of quantification (LLOQ) as 10 : 1, at which accuracy and standard deviation were within 20% as per ICH guidelines [35].

#### 2.7.3. Accuracy and Precision

Intraday accuracy and precision were performed for QCs of the analytes at concentrations of 0.25, 0.5, 1, and 2 *μ*g·mL^−1^ for clopidogrel bisulfate, 0.5, 1, 2, and 4 *μ*g·mL^−1^ for its carboxylic acid metabolite, and 0.2, 0.3, 1, and 2.5 *μ*g·mL^−1^ for atorvastatin in replicate (*n* = 3). Interday accuracy and precision were achieved from the same QCs on three different days. QCs were analyzed using calibration curves. Accuracy (expressed as recovery) and precision (expressed as relative standard deviation) should not deviate by ±15% of the nominal concentration.

#### 2.7.4. Stability

*(1) Short-Term Stability*. Stability of clopidogrel carboxylic acid metabolite and atorvastatin in human plasma was evaluated at concentrations of 0.12 and 4 *μ*g·mL^−1^ for clopidogrel carboxylic acid metabolite and 0.5 and 2.5 *μ*g·mL^−1^ for atorvastatin in three replicates for each concentration at ambient temperature for 6 hours (short-term stability) and then processed and analyzed identical to samples procedure. The stability of the analytes was determined against a freshly prepared stock solution. The stability was consistent if the deviation in concentration was within ±15%.

*(2) Long-Term Stability*. Three aliquots of low and high unprocessed QCs (0.12 and 4 *μ*g·mL^−1^ for clopidogrel carboxylic acid metabolite and 0.5 and 2.5 *μ*g·mL^−1^ for atorvastatin) were kept at −20°C for 30 days. The concentrations of the analytes after each storage period were calculated using a calibration curve, obtained from freshly prepared samples in the same analytical run. The samples were considered stable if the percentage change in the concentration was within ±15%.

#### 2.7.5. Robustness

The method robustness was performed by evaluating the influence of small changes in chromatographic conditions, such as flow rate (±0.1 mL·min^−1^) and pH (±0.2 units). The flow rate of the mobile phase was 1 mL·min^−1^ and modified from 0.9 to 1.1 mL·min^−1^. The pH of the buffer solution was 2.6 and modified to 2.8.

#### 2.7.6. System Suitability Tests (SST)

The most important SST parameters which were investigated for the HPLC analysis were resolution, retention time, column efficiency (N), and tailing factor (T). SST limits for HPLC method were settled according to international guidelines recommendations (resolution > 2.0, tailing factor ≤ 2.0, and plate count > 2000). Data from six replicate injections were used for SST and summarized. The chromatographic system was brought into compliance with the system suitability requirements.

#### 2.7.7. Degradation Studies

According to ICH Q2B Validation of Analytical Procedures: Methodology, forced degradation studies were performed under various stress conditions with 1 M HCl, 1 M NaOH, and 5% H_2_O_2_ at 60°C for three hours.

*(1) Procedure for Preparation of Degradation Products*. Stock solutions of clopidogrel bisulfate and atorvastatin calcium were prepared at the concentration of 100 *μ*g·mL^−1^. 10 mL of clopidogrel bisulfate and atorvastatin calcium stock 20 solution were individually mixed with 10 mL of 1 M HCl, 1 M NaOH, and 5% H_2_O_2_. The solution mixtures were heated at 60°C for three hours, neutralized with 1 M NaOH and 1 M HCl for the acidic and alkaline degradation, respectively. From the resultant solutions 1 mL was diluted with methanol to the final concentration of 25 *μ*g·mL^−1^ and 20 *μ*L was injected into the system.

### 2.8. *In Vivo* Application

The method has been applied in simultaneous quantification of clopidogrel, its carboxylic acid metabolite, and atorvastatin in patient's plasma following oral administration of 75 mg clopidogrel and 20, 40, and 80 mg atorvastatin during their maintenance therapy with both drugs. The study protocol was approved by Ethical Committee of Medicine and Pharmacy University from Craiova, Romania, and patients had signed an informed consent. The studies were performed according to the Declaration of Helsinki and Good Clinical Practice guidelines [36]. Three patients (two males and one female 80, 60, and 53 years old) were included in the studies. Blood samples were collected before and at 0.25, 0.5, 1, 3, 6, 9, and 12 h after administration of the drug. The aliquot of 5 mL of blood was drawn into an EDTA tube and plasma was separated by centrifugation for 10 min at 4500 rpm and stored at −20°C.

### 2.9. Pharmacokinetic Calculations

Data from seven sampling times within 12 h after multiple oral drug intakes were used to calculate pharmacokinetic parameters: absorption rate constant, *Ka*, time of peak concentration, *t*_max_, maximum plasma concentration, *C*_max_, elimination rate constant, *Ke*, area under the concentration-time curve from time zero to time *t* (AUC_0-*t*_), and area under the concentration-time curve from time zero to time infinity (AUC_0-*∞*_). Pharmacokinetic parameters are derived from data by mathematical calculations [37]. The overall absorption process is considered to be a single first-order process, One Compartment Pharmacokinetic Model. The slope of the plasma drug concentration versus time plot is –*Ke*/2.303. The terminal slope is then back-extrapolated to the concentration axis. Absorption rate constant, *Ka*, was estimated by applying the method of residuals. The elimination half-time (*t*_1/2_) was estimated from ln⁡2/*Ke*. Time of peak concentration, *t*_max_, was derived by setting the rate of change of *C*_*p*_ (plasma concentration) versus time, *dC*_*p*_/*dt*, to zero: *t*_max_ = ln⁡(*Ka*/*Ke*)/(*Ka* − *Ke*). Area under the concentration-time curve from time zero to time *t* (AUC_0-*t*_), where *t* is the last measurable time, was calculated by trapezoidal rule. Area under the concentration-time curve from time zero to time infinity (AUC_0-*∞*_) was calculated totaling AUC_0-*t*_ to *C*_last_/*Ke*, where *C*_last_ is the last measurable concentration.

## 3. Results and Discussion

### 3.1. Method Validation

#### 3.1.1. Linearity

The calibration plot of peak area against concentration was linear in the range investigated, 0.008–2 *μ*g·mL^−1^ for clopidogrel, 0.01–4 *μ*g·mL^−1^ for its carboxylic acid metabolite, and 0.005–2.5 *μ*g·mL^−1^ for atorvastatin. The regression equations and correlation coefficient were as follows: *y* = 0.3789*x* + 0.0004, *r* ^2^ = 0.9998 for clopidogrel bisulfate, *y* = 0.2227*x* + 0.0158, *r* ^2^ = 0.9995 for clopidogrel carboxylic acid metabolite, and *y* = 0.3404*x* + 0.0017, *r* ^2^ = 0.9994 for atorvastatin, respectively, with ibuprofen as internal standard.

#### 3.1.2. Limit of Detection and Quantification

The LOD and LLOQ for clopidogrel bisulfate were 0.003 *μ*g·mL^−1^ and 0.008 *μ*g·mL^−1^, while for its carboxylic acid metabolite 0.004 *μ*g·mL^−1^ and 0.01 *μ*g·mL^−1^. For atorvastatin the LOD and LLOQ were 0.002 *μ*g·mL^−1^ and 0.005 *μ*g·mL^−1^, respectively.

#### 3.1.3. Accuracy and Precision

Accuracy and precision results are summarized in Table 2. For intraday assay, the accuracy for QS samples expressed as recovery was in the range of 96–98% for clopidogrel bisulfate, 94–98% for its metabolite, and 90–98% for atorvastatin, respectively, and for interday assay 96-97% for clopidogrel, 94–98% for its metabolite, and 90–99% for atorvastatin. In the case of intraday precision, the relative standard deviation (RSD%) was in the range 2.03–4.17% for clopidogrel bisulfate, 1.02–4.26% for its metabolite, and 1.03–5.55% for atorvastatin. For interday assay, the results showed ranges of 2.08–8.33% for clopidogrel bisulfate, 1.04–2.12% for its metabolite, and 0.40–6.90% for atorvastatin.

#### 3.1.4. Stability

The results obtained during short- and long-term stability for clopidogrel carboxylic acid metabolite and atorvastatin are indicated in Table 3. The recovery for clopidogrel carboxylic acid metabolite ranged between 92 and 97% at the concentrations of 0.12 and 4 *μ*g·mL^−1^ during short-term tests and 92 and 96% during long-term stability study at the same concentrations. The recovery for atorvastatin in short-term study was in the range of 94–96% at the concentrations of 0.5 and 2.5 *μ*g·mL^−1^ and 92–96% for long-term stability study.

#### 3.1.5. Robustness and System Suitability Test

The assay variability of clopidogrel bisulfate and atorvastatin calcium is summarized in Table 4 in deliberate varied chromatographic conditions (flow rate and pH). System suitability test results are presented in Table 5.

#### 3.1.6. Specificity

**Figure 2:**
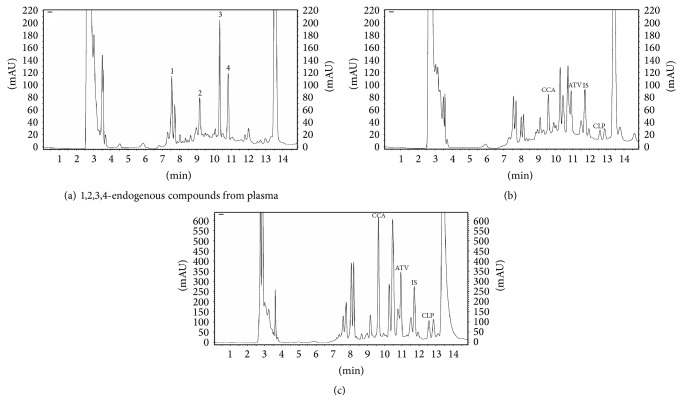
Chromatograms of blank plasma (a); drug-free human plasma spiked with clopidogrel carboxylic acid metabolite (CCA with *t*_*R*_ = 9.663 min), atorvastatin (ATV with *t*_*R*_ = 10.998 min), IS-ibuprofen (2 *μ*g·Ml^−1^, *t*_*R*_ = 11.802 min), and clopidogrel bisulfate (CLP with *t*_*R*_ = 12.682 min) (b); plasma sample from a volunteer who received 75 mg clopidogrel and 40 mg atorvastatin at 40 minutes after dose under optimized gradient method at flow rate of 1 mL·min^−1^ (c).

The gradient method allowed optimum separation of analytes from matrix interference. Drug-free human plasma for ten lots was analyzed for endogenous presence. Two extracted endogenous compounds had retention times of 2.673 min and 13.582 min, respectively, but they did not elute at the same time as the analytes. No significant interfering peaks were observed at the retention times of analytes. The retention times of clopidogrel carboxylic acid metabolite, atorvastatin, IS-ibuprofen, and clopidogrel bisulfate were 9.663, 10.998, 11.802, and 12.682 min. The chromatograms of blank plasma, spiked plasma and from a patient who received 75 mg clopidogrel and 40 mg atorvastatin at 40 min after dose are presented in Figure 2.

#### 3.1.7. Degradation Studies

**Figure 3:**
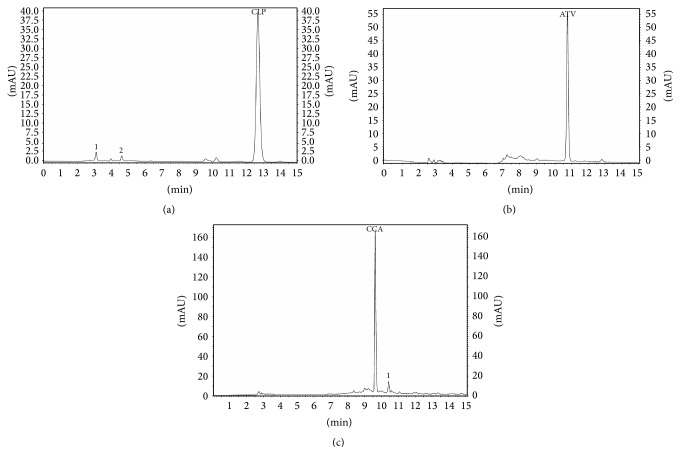
Chromatograms of clopidogrel bisulfate, its carboxylic acid metabolite, and atorvastatin calcium 25 *μ*g·mL^−1^ in optimized mobile phase (1,2-impurities).

**Figure 4:**
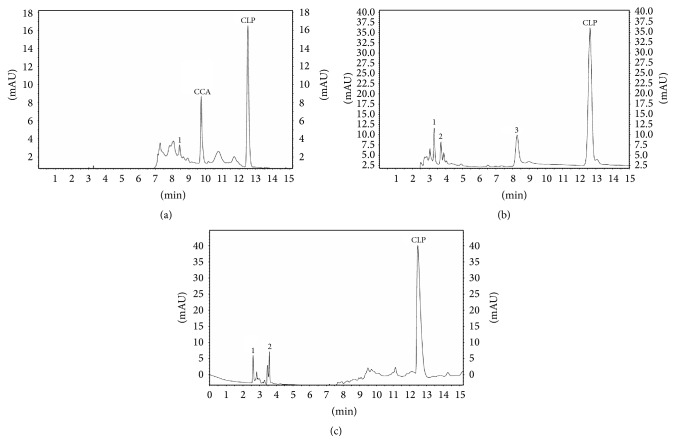
Chromatograms of acid (a), basic (b), and oxidative (c) stress degradation samples of clopidogrel bisulfate (1,2,3-degradation products).

**Figure 5:**
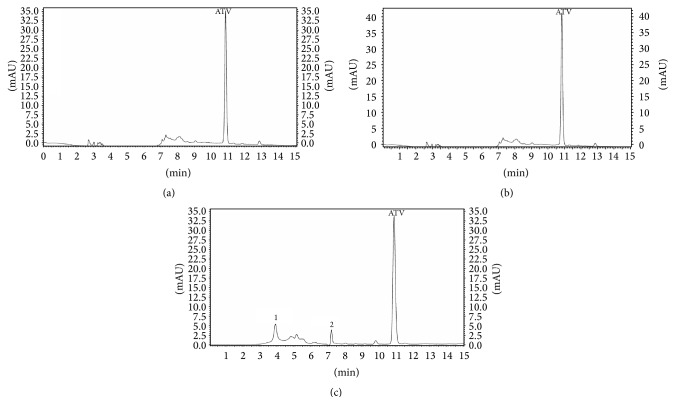
Chromatograms of acid (a), basic (b), and oxidative (c) stress degradation samples of atorvastatin calcium (1,2-degradation products).

The chromatograms of pure clopidogrel bisulfate and atorvastatin calcium are presented in Figure 3. Significant degradation was observed under acidic (38%, *t*_*R*_ = 9.75 min), basic (30%, *t*_*R*_ = 8.30), and oxidative (20%) conditions for clopidogrel bisulfate presented in Figure 4, while for atorvastatin calcium no significant degradation under acidic and basic conditions was observed (Figure 5).  Table 6 summarizes the analytes recovery after stress degradation and the retention times of the degraded products to clopidogrel bisulfate and atorvastatin calcium. Well separation of degradation products from parent peak shows that the method is stability indicating.

### 3.2. *In Vivo* Studies

**Figure 6:**
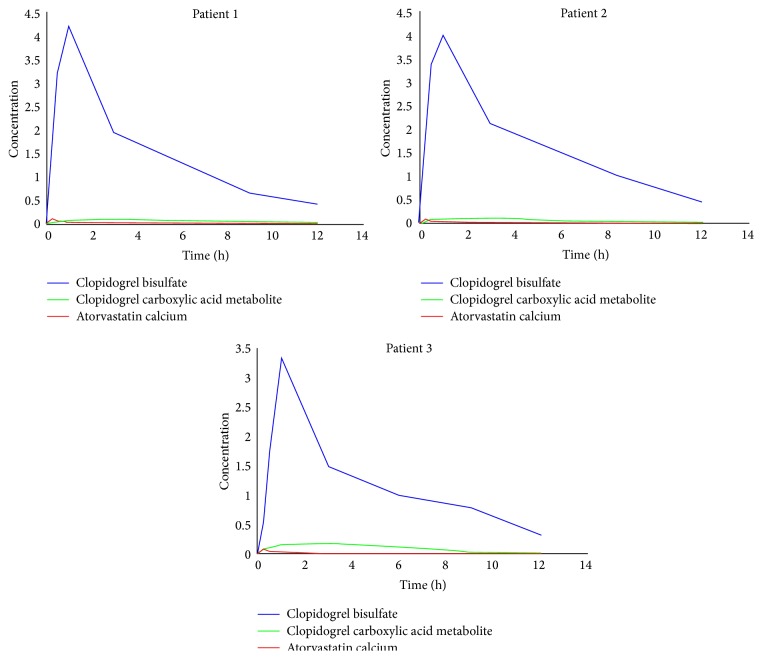
Plasma levels of clopidogrel, its carboxylic acid metabolite, and atorvastatin versus time following administration of 75 mg clopidogrel and 20 mg atorvastatin daily (patient 1), 75 mg clopidogrel and 40 mg atorvastatin daily (patient 2), and 75 mg clopidogrel and 80 mg atorvastatin daily (patient 3).

The applicability of the method has been demonstrated in a pharmacokinetic study to patients undergoing antiplatelet therapy with 75 mg clopidogrel and atorvastatin in different concentrations (20, 40, and 80 mg). Pharmacokinetic parameters are summarized in Table 7. The mean plasma concentration-time curves of clopidogrel, its carboxylic acid metabolite, and atorvastatin in three patients with different dosage regimen are shown in Figure 6. Plasma levels of the prodrug clopidogrel beyond 3 h from administration are below the quantification limit (LLOQ 0.008 *μ*g·mL^−1^) of the gradient method and plasma levels of its carboxylic acid metabolite are much higher than those of clopidogrel. Atorvastatin reached low plasmatic concentration due to its low bioavailability but presented an increased half-life time based on its multiple metabolites and plasmatic protein binding in the percentage of 98%. These results are in direct correlation with patients intraindividual variability also assuming the long period of time of multidrug therapy.

Effects of atorvastatin on clopidogrel pharmacokinetics were modest for the patients included in the study in order to stop their coadministration based on a drug-drug interaction. It is worth mentioning that little changes in the pharmacokinetics may induce major cardiovascular effects in patients with high risk. Despite the long period of time since clopidogrel was approved on the market and the large number of clinical studies, the pharmacokinetics of this drug is still fully unrevealed by the scientific community.

## 4. Conclusions

A sensitive, specific, and reproducible RP-HPLC method was developed and validated for the simultaneous analysis of clopidogrel bisulfate and its carboxylic acid metabolite along with atorvastatin in clinical samples. The method was successfully applied for determination of the analytes levels in plasma of patients treated with 75 mg clopidogrel and 20, 40, and 80 mg atorvastatin daily, with significant sensitivity for clopidogrel carboxylic acid metabolite (LLOQ = 0.01 *μ*g·mL^−1^). Extraction of the analytes from the serum has been achieved in a single step by protein precipitation with acetonitrile, with improved recovery in the range of 96–98% for clopidogrel, 94–98% for clopidogrel carboxylic acid metabolite, and 90–99% for atorvastatin, respectively. The gradient program has been proposed in order to overcome the matrix effect at the beginning of the analysis and close retention times of clopidogrel and atorvastatin.

## Supplementary Material

The supplementary data contains chromatograms of plasma spiked with respective standards of clopidogrel bisulfate, its carboxylic acid metabolite, atorvastatin and ibuprofen (IS.) at 50 μg·mL^−1^, obtained under the optimized chromatographic method. The chromatograms are presented in Figure S1 in order to detect any peaks interfering the target compounds. The chromatograms indicate no significant interference from impurities at the retention times of the analytes ( t_R_=9.64 min for clopidogrel carboxylic acid metabolite, 10.94 min for atorvastatin, 12.50 min for clopidogrel bisulfate and 11.90 min for ibuprofen as IS). Linearity of the calibration curves was estimated for the ratio of the peak area of clopidogrel bisulfate, its carboxylic acid metabolite and atorvastatin to that of the internal standard, ibuprofen (Figure S2), covering the range of 0.008-2 μg·mL^−1^ for clopidogrel bisulfate, 0.01-4 μg·mL^−1^ for its carboxylic acid metabolite and 0.005-2.5 μg·mL^−1^ for atorvastatin. The equations of calibration curves presented in Table S1 were used to calculate concentrations of clopidogrel bisulfate, its carboxylic acid metabolite and atorvastatin in patients´ plasma. The correlation coefficients were also calculated and presented in Table S1.
Data from seven sampling times within 12 h after multiple oral drug intakes were used to calculate pharmacokinetic parameters: absorption rate constant, k_a_, time of peak concentration, tmax, maximum plasma concentration, C_max_, eliminate rate constant, k_e_, Area under the concentration-time curve from time zero to time t (AUC_0-t_) and Area under the concentration-time curve from time zero to time infinity (AUC_0-∞_).
The plasma levels of the analytes were used to calculate pharmacokinetic parameters by mathematical calculations. C_max_ and t_max_ were read from individual analyte concentration-time curve. The elimination half-life( t_1/2_) was estimated from ln2/ke, where k_e_ is the elimination rate constant. Area under the concentration-time curve from time zero to time t (AUC_0-t_), where t is the last measurable time, was calculated by trapezoidal rule. The total area under the concentration-time curve (AUC_0-∞_).was estimated by trapezoidal rule with extrapolation to infinity using *Ct/ke*

## Figures and Tables

**Table 1:** Gradient program for simultaneous determination of clopidogrel, its carboxylic acid metabolite, and atorvastatin.

Time	Solvent A	Solvent B	Solvent C
(min)	(Buffer solution)	(ACN)	(CH_3_OH)
0.01	90	10	0
2.00	90	10	0
3.00	60	40	0
4.00	30	50	20
8.00	30	50	20
10.00	30	40	30
18.00	30	50	20
19.00	90	10	0
20.00	90	10	0

**Table 2:** Intraday and interday precision and accuracy of the method.

Analyte	Period of analysis	Nominal concentration *μ*g·mL^−1^	Mean concentration found *μ*g·mL^−1^	Recovery %	RSD %	*n*
CLP	Intraday	0.25	0.24 ± 0.01	96	4.17	3
		0.50	0.49 ± 0.02	98	4.08	3
		1	0.97 ± 0.02	97	2.06	3
		2	1.97 ± 0.04	98	2.03	3
	Interday	0.25	0.24 ± 0.02	96	8.33	3
		0.50	0.48 ± 0.01	96	2.08	3
		1	0.96 ± 0.02	96	2.08	3
		2	1.94 ± 0.05	97	2.57	3

CCA	Intraday	0.5	0.47 ± 0.02	94	4.26	3
		1	0.95 ± 0.01	95	1.05	3
		2	1.96 ± 0.02	98	1.02	3
		4	3.87 ± 0.08	97	2.07	3
	Interday	0.50	0.47 ± 0.01	94	2.12	3
		1	0.96 ± 0.01	96	1.04	3
		2	1.95 ± 0.03	98	1.54	3
		4	3.88 ± 0.08	97	2.06	3

ATV	Intraday	0.20	0.18 ± 0.01	90	5.55	3
		0.3	0.28 ± 0.01	93	3.57	3
		1	0.97 ± 0.01	97	1.03	3
		2.5	2.44 ± 0.03	98	1.23	3
	Interday	0.20	0.18 ± 0.01	90	5.56	3
		0.30	0.29 ± 0.02	97	6.90	3
		1	0.98 ± 0.05	98	5.10	3
		2.5	2.47 ± 0.01	99	0.40	3

CLP: clopidogrel bisulfate; CCA: clopidogrel carboxylic acid metabolite; ATV: atorvastatin.

**Table 3:** Results of stability tests for clopidogrel carboxylic acid metabolite and atorvastatin.

Analyte	Stability	Nominal concentration *μ*g·mL^−1^	Mean found concentration *μ*g·mL^−1^	Recovery %	RSD %	*n*
CCA	Short-term (6 h at room temperature)	0.12	0.11 ± 0.01	92	9.09	3
		4	3.86 ± 0.15	97	3.89	3
	Long-term (30 days at room temperature)	0.12	0.11 ± 0.01	92	9.08	3
		4	3.85 ± 0.06	96	1.56	3

ATV	Short-term (6 h at room temperature)	0.5	0.47 ± 0.01	94	2.13	3
		2.5	2.41 ± 0.02	96	0.83	3
	Long-term (30 days at room temperature)	0.5	0.46 ± 0.05	92	10.87	3
		2.5	2.41 ± 0.01	96	0.42	3

**Table 4:** Robustness results for the RP-HPLC method.

Parameters	CCA		ATV		CLP	
	Retention time (min) ± SD	% RSD	Retention time (min) ± SD	% RSD	Retention time (min) ± SD	% RSD
Flow rate mL·min^−1^						
0.9	9.75 ± 0.02	0.26	11.03 ± 0.01	0.61	12.96 ± 0.03	1.58
1	9.66 ± 0.01	0.31	10.99 ± 0.02	0.66	12.68 ± 0.01	1.57
1.1	9.44 ± 0.03	0.20	10.90 ± 0.03	0.52	12.48 ± 0.03	0.95
pH						
2.6	9.66 ± 0.02	0.36	10.99 ± 0.01	0.66	12.68 ± 0.01	1.55
2.8	9.65 ± 0.01	0.20	10.93 ± 0.03	0.39	12.50 ± 0.03	0.96

*n* = 6: number of replicates.

**Table 5:** System suitability test results for clopidogrel bisulfate, its carboxylic acid metabolite, and atorvastatin calcium by the proposed LC method.

Parameter	Obtained value			Reference value
	CCA	ATV	CLP	
Analyte				
Retention time (min)	9.66	10.99	12.68	
Tailing factor (*T*)	0.94	1.12	1.5	≤2.0
Plate count (*N*)	14763	15935	18340	>2000
Resolution (*R*_*s*_)	8.75		8.82	>2.00

CLP: clopidogrel bisulfate; CCA: clopidogrel carboxylic acid metabolite; ATV: atorvastatin; SD: standard deviation; RSD: relative standard deviation.

**Table 6:** Results of degradation studies on clopidogrel bisulfate and atorvastatin calcium.

Condition	Time (h)	Temperature (°C)	CLP (recovery %)	ATV (recovery %)	*t*_*R*_ for CLP degraded products	*t*_*R*_ for ATV degraded products
1 M HCl	3	60	62	89	*t*_1_ = 8.39 *t*_2_ = 9.75	—

1 M NaOH	3	60	70	73	*t*_1_ = 3.31 *t*_2_ = 3.72 *t*_3_ = 8.30	—

5% H_2_O_2_	3	60	80	67	*t*_1_ = 2.64 *t*_2_ = 3.54	*t*_1_ = 3.93 *t*_2_ = 7.20

CLP: clopidogrel bisulfate; ATV: atorvastatin.

**Table 7:** Pharmacokinetic parameters for clopidogrel bisulfate, its carboxylic acid metabolite, and atorvastatin calcium.

	Elimination rate constant	Half-life *t*_1/2_	*T*_max⁡_	*C*_max⁡_	AUC_0-*t*_	AUC_0-*∞*_
	*Ke* (h^−1^)	(h)	(h)	(*μ*g·mL^−1^)	(*μ*g*∗*h/mL)	(*μ*g*∗*h·mL^−1^)
Patient 1 (clopidogrel 75 mg, atorvastatin 20 mg)						
CLP	0.81	0.85	0.25	0.1	0.85	0.85
CCA	0.15	4.51	1	4.2	17.79	20.46
ATV20	0.08	8.15	3	0.09	0.67	1.03

Patient 2 (clopidogrel 75 mg, atorvastatin 40 mg)						
CLP	0.58	1.20	0.25	0.08	0.63	0.63
CCA	0.23	3	1.24	4.77	19.61	21.55
ATV40	0.08	8.37	2.52	0.15	0.91	0.96

Patient 3 (clopidogrel 75 mg, atorvastatin 80 mg)						
CLP	1.02	0.68	0.25	0.07	0.65	0.65
CCA	0.13	5.21	1	3.32	14.38	15.45
ATV80	0.07	9.32	3.06	0.19	1.32	1.36

## References

[1] Hamm C. W., Bassand J.-P., Agewall S. (2011). ESC guidelines for the management of acute coronary syndromes in patients presenting without persistent ST-segment elevation. *European Heart Journal*.

[2] Amsterdam E. A., Wenger N. K., Brindis R. G. (2014). 2014 AHA/ACC guideline for the management of patients with non-ST-elevation acute coronary syndromes. *Circulation*.

[3] Pereillo J.-M., Maftouh M., Andrieu A. (2002). Structure and stereochemistry of the active metabolite of clopidogrel. *Drug Metabolism and Disposition*.

[4] Kazui M., Nishiya Y., Ishizuka T. (2010). Identification of the human cytochrome P450 enzymes involved in the two oxidative steps in the bioactivation of clopidogrel to its pharmacologically active metabolite. *Drug Metabolism and Disposition*.

[5] Bouman H. J., Schömig E., van Werkum J. W. (2011). Paraoxonase-1 is a major determinant of clopidogrel efficacy. *Nature Medicine*.

[6] Igel M., Sudhop T., Bergmann K. (2002). Pharmacology of 3-hydroxy-3-methylglutaryl-coenzyme A reductase inhibitors (statins), including rosuvastatin and pitavastatin. *The Journal of Clinical Pharmacology*.

[7] Malhotra H. S., Goa K. L. (2001). Atorvastatin: an updated review of its pharmacological properties and use in dyslipidaemia. *Drugs*.

[8] Lea A. P., McTavish D. (1997). Atorvastatin. A review of its pharmacology and therapeutic potential in the management of hyperlipidaemias. *Drugs*.

[9] Rouini M.-R., Ardakani Y. H., Foroumadi A., Lavasani H., Hakemi L. (2009). Sensitive quantification of carboxylic acid metabolite of clopidogrel in human plasma by LC with UV detection. *Chromatographia*.

[10] Bahrami G., Mohammadi B., Sisakhtnezhad S. (2008). High-performance liquid chromatographic determination of inactive carboxylic acid metabolite of clopidogrel in human serum: application to a bioequivalence study. *Journal of Chromatography B: Analytical Technologies in the Biomedical and Life Sciences*.

[11] Singh S. S., Sharma K., Barot D., Mohan P. R., Lohray V. B. (2005). Estimation of carboxylic acid metabolite of clopidogrel in Wistar rat plasma by HPLC and its application to a pharmacokinetic study. *Journal of Chromatography B*.

[12] Ksycinska H., Rudzki P., Bukowska-Kiliszek M. (2006). Determination of clopidogrel metabolite (SR26334) in human plasma by LC–MS. *Journal of Pharmaceutical and Biomedical Analysis*.

[13] Mitakos A., Panderi I. (2004). Determination of the carboxylic acid metabolite of clopidogrel in human plasma by liquid chromatography-electrospray ionization mass spectrometry. *Analytica Chimica Acta*.

[14] Karaźniewicz-Łada M., Danielak D., Tezyk A., zaba C., Tuffal G., Główka F. (2012). HPLC-MS/MS method for the simultaneous determination of clopidogrel, its carboxylic acid metabolite and derivatized isomers of thiol metabolite in clinical samples. *Journal of Chromatography B*.

[15] Silvestro L., Gheorghe M. C., Tarcomnicu I. (2010). Development and validation of an HPLC-MS/MS method to determine clopidogrel in human plasma. Use of incurred samples to test back-conversion. *Journal of Chromatography B*.

[16] Reddy S R., Rao.Divi K., chandiran I. S., Jayaveera K. N., Naidu Y. K., Reddy M. P. K. (2010). Development and validation of high-throughput liquid chromatography-tandem mass spectrometric method for simultaneous quantification of Clopidogrel and its metabolite in human plasma. *Journal of Chromatography B: Analytical Technologies in the Biomedical and Life Sciences*.

[17] Di Girolamo G., Czerniuk P., Bertuola R., Keller G. A. (2010). Bioequivalence of two tablet formulations of clopidogrel in healthy Argentinian volunteers: a single-dose, randomized-sequence, open-label crossover study. *Clinical Therapeutics*.

[18] Small D. S., Payne C. D., Kothare P. (2010). Pharmacodynamics and pharmacokinetics of single doses of prasugrel 30 mg and clopidogrel 300 mg in healthy Chinese and white volunteers: an open-label trial. *Clinical Therapeutics*.

[19] Zou J.-J., Fan H.-W., Guo D.-Q. (2009). Simultaneous determination of clopidogrel and its carboxylic acid metabolite (SR26334) in human plasma by LC-ESI-MS-MS: application to the therapeutic drug monitoring of clopidogrel. *Chromatographia*.

[20] Robinson A., Hillis J., Neal C., Leary A. C. (2007). The validation of a bioanalytical method for the determination of clopidogrel in human plasma. *Journal of Chromatography B*.

[21] Furlong M. T., Savant I., Yuan M. (2013). A validated HPLC-MS/MS assay for quantifying unstable pharmacologically active metabolites of clopidogrel in human plasma: application to a clinical pharmacokinetic study. *Journal of Chromatography B*.

[22] Peer C. J., Spencer S. D., VanDenBerg D. A. H., Pacanowski M. A., Horenstein R. B., Figg W. D. (2012). A sensitive and rapid ultra HPLC-MS/MS method for the simultaneous detection of clopidogrel and its derivatized active thiol metabolite in human plasma. *Journal of Chromatography B*.

[23] Tuffal G., Roy S., Lavisse M. (2011). An improved method for specific and quantitative determination of the clopidogrel active metabolite isomers in human plasma. *Thrombosis and Haemostasis*.

[24] Takahashi M., Pang H., Kawabata K., Farid N. A., Kurihara A. (2008). Quantitative determination of clopidogrel active metabolite in human plasma by LC–MS/MS. *Journal of Pharmaceutical and Biomedical Analysis*.

[25] Bahrami G., Mohammadi B., Mirzaeei S., Kiani A. (2005). Determination of atorvastatin in human serum by reversed-phase high-performance liquid chromatography with UV detection. *Journal of Chromatography B*.

[26] Hassan J., Bahrani S. H. (2014). Determination of atorvastatin in human serum by salting out assisted solvent extraction and reversed-phase high-performance liquid chromatography-UV detection. *Arabian Journal of Chemistry*.

[27] Partani P., Verma S. M., Gurule S., Khuroo A., Monif T. (2014). Simultaneous quantitation of atorvastatin and its two active metabolites in human plasma by liquid chromatography/(–) electrospray tandem mass spectrometry. *Journal of Pharmaceutical Analysis*.

[28] Hermann M., Christensen H., Reubsaet J. L. E. (2005). Determination of atorvastatin and metabolites in human plasma with solid-phase extraction followed by LC–tandem MS. *Analytical and Bioanalytical Chemistry*.

[29] Ma L., Dong J., Chen X. J., Wang G. J. (2007). Development and validation of atorvastatin by LC-ESI-MS and application in bioequivalence research in healthy Chinese volunteers. *Chromatographia*.

[30] Zhou Y., Li J., He X. (2013). Development and validation of a liquid chromatography-tandem mass spectrometry method for simultaneous determination of amlodipine, atorvastatin and its metabolites ortho-hydroxy atorvastatin and para-hydroxy atorvastatin in human plasma and its application in a bioequivalence study. *Journal of Pharmaceutical and Biomedical Analysis*.

[31] Yacoub M., Abu Awwad A., Alawi M., Arafat T. (2013). Simultaneous determination of amlodipine and atorvastatin with its metabolites; *ortho* and *para* hydroxy atorvastatin; in human plasma by LC–MS/MS. *Journal of Chromatography B*.

[32] Polagani S. R., Pilli N. R., Gajula R., Gandu V. (2013). Simultaneous determination of atorvastatin, metformin and glimepiride in human plasma by LC–MS/MS and its application to a human pharmacokinetic study. *Journal of Pharmaceutical Analysis*.

[33] Shah Y., Iqbal Z., Ahmad L. (2011). Simultaneous determination of rosuvastatin and atorvastatin in human serum using RP-HPLC/UV detection: method development, validation and optimization of various experimental parameters. *Journal of Chromatography B*.

[34] Nováková L., Vlčková H., Šatínský D. (2009). Ultra high performance liquid chromatography tandem mass spectrometric detection in clinical analysis of simvastatin and atorvastatin. *Journal of Chromatography B*.

[35] ICH Validation of analytical procedures and methodology.

[36] International Conference on Harmonisation of Technical Requirements for Registration of Pharmaceuticals for Human Use (1996). *ICH Harmonised Tripartite Guideline. Guideline for Good Clinical Practice E6(R10) Current Step 4 Version*.

[37] Hedaya M. A. (2012). Extravascular routes of drug administration. *Basic Pharmacokinetics*.

