# Supplementary material for: Development and Validation of an HPLC Method for Simultaneous Quantification of Clopidogrel Bisulfate, Its Carboxylic Acid Metabolite, and Atorvastatin in Human Plasma: Application to a Pharmacokinetic Study
Source: J Anal Methods Chem. 2015 Dec 29;2015:892470. doi: 10.1155/2015/892470 (PMC4709620; doi:10.1155/2015/892470)
Supplement: Supplementary file 1 — The supplementary data contains chromatograms of plasma spiked with respective standards of clopidogrel bisulfate, its carboxylic acid metabolite, atorvastatin and ibuprofen (IS.) at 50 μg·mL−1, obtained under the optimized chromatographic method. The chromatograms are presented in Figure S1 in order to detect any peaks interfering the target compounds. The chromatograms indicate no significant interference from impurities at the retention times of the analytes ( tR=9.64 min for clopidogrel carboxylic acid metabolite, 10.94 min for atorvastatin, 12.50 min for clopidogrel bisulfate and 11.90 min for ibuprofen as IS). Linearity of the calibration curves was estimated for the ratio of the peak area of clopidogrel bisulfate, its carboxylic acid metabolite and atorvastatin to that of the internal standard, ibuprofen (Figure S2), covering the range of 0.008-2 μg·mL−1 for clopidogrel bisulfate, 0.01-4 μg·mL−1 for its carboxylic acid metabolite and 0.005-2.5 μg·mL−1 for atorvastatin. The equations of calibration curves presented in Table S1 were used to calculate concentrations of clopidogrel bisulfate, its carboxylic acid metabolite and atorvastatin in patients´ plasma. The correlation coefficients were also calculated and presented in Table S1. Data from seven sampling times within 12 h after multiple oral drug intakes were used to calculate pharmacokinetic parameters: absorption rate constant, ka, time of peak concentration, tmax, maximum plasma concentration, Cmax, eliminate rate constant, ke, Area under the concentration-time curve from time zero to time t (AUC0-t) and Area under the concentration-time curve from time zero to time infinity (AUC0-∞). The plasma levels of the analytes were used to calculate pharmacokinetic parameters by mathematical calculations. Cmax and tmax were read from individual analyte concentration-time curve. The elimination half-life( t1/2) was estimated from ln2/ke, where ke is the elimination rate constant. Area under the concentration-time [file 892470.f1.pdf]

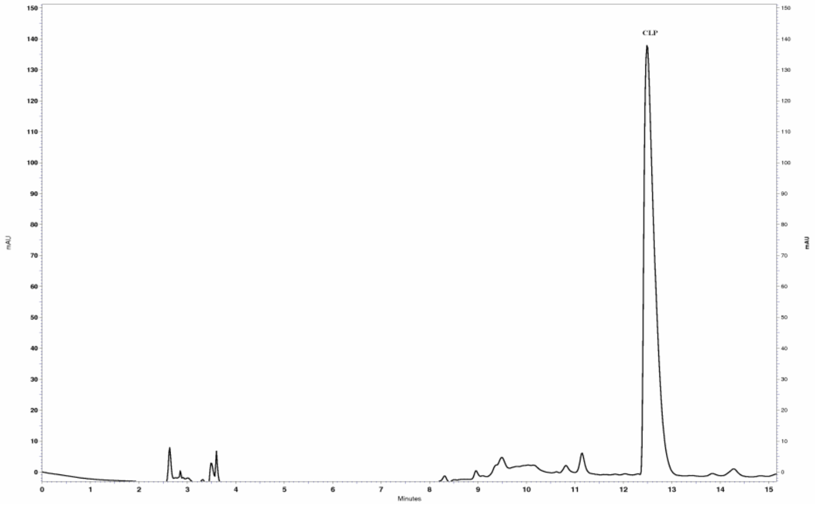
CLP
mAU
Minutes

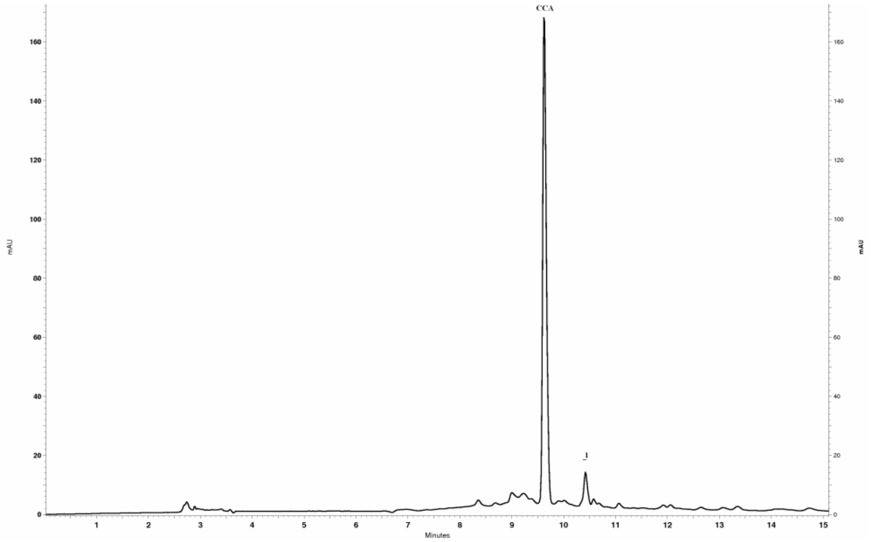
CCA
mAU
Minutes

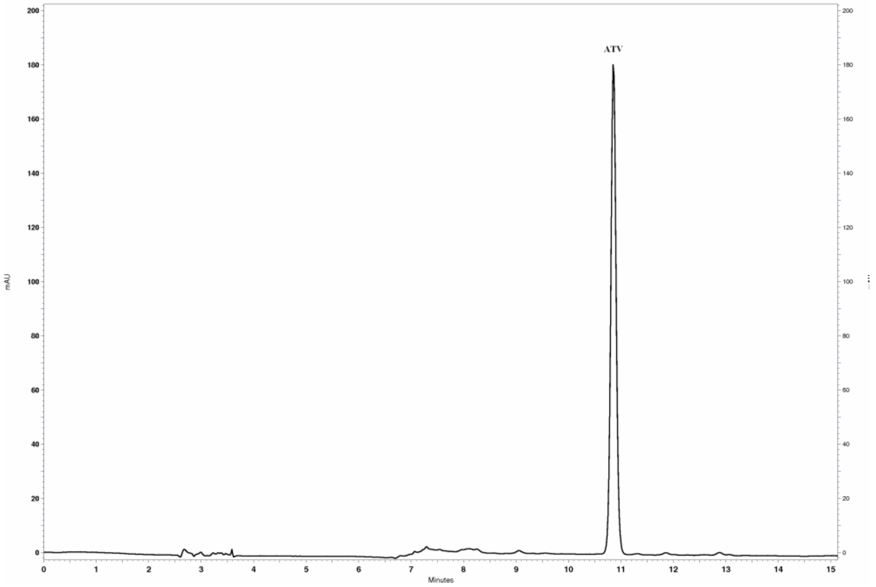
ATV
mAU
Minutes

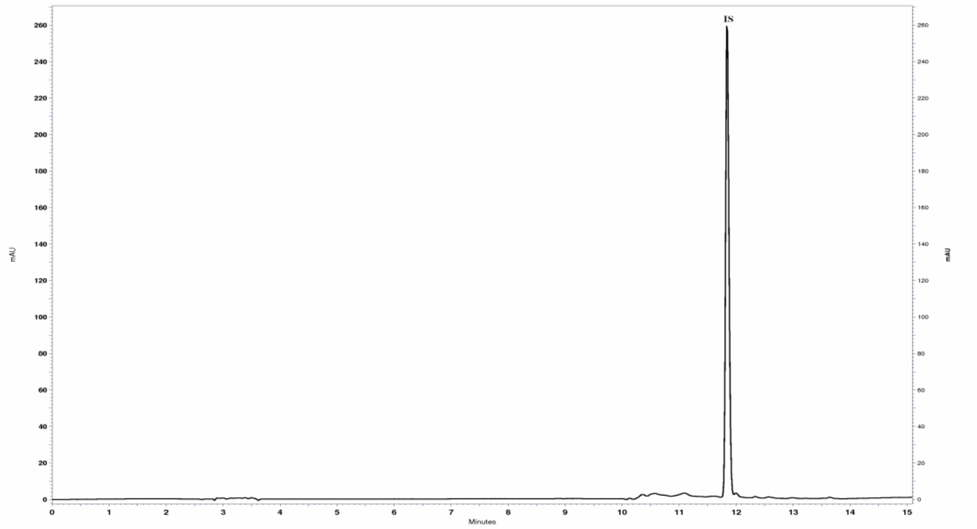

Figure S1 Chromatograms of clopidogrel bisulfate, its carboxylic acid metabolite, atorvastatin calcium and ibuprofen (IS) stock solution under the optimized chromatographic method

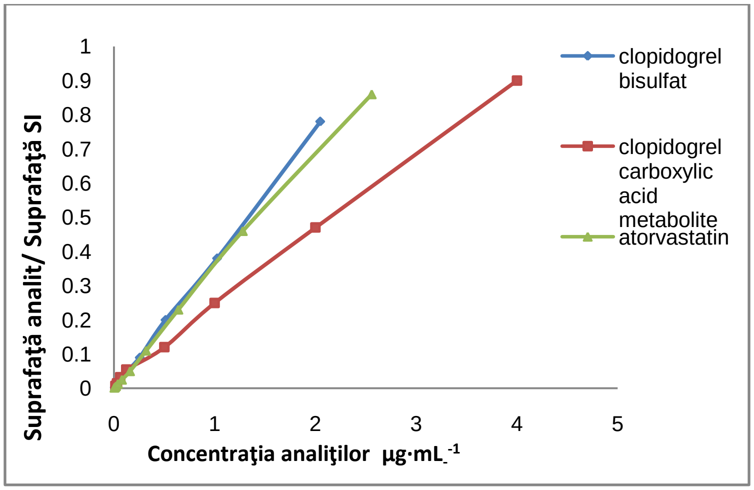

Figure S2 *Calibation curves of clopidogrel bisulfate, its carboxylic acid metabolite and atorvastatin*

Table S1 Results of calibration curves (n=3)

Method	Analyte	Range (μg/mL)	Regression ecuation	r^2
HPLC gradient method, I.S.- ibuprofen (2μg/mL)	Clopidogrel bisulfate	0.008-2	y=0.3789 x+0.0004	0.9998
	Clopidogrel carboxylic acid	0.01-4	Y=0.2227 x+0.0158	0.9995
	Atorvastatin	0.005-2.5	Y=0.3404 x+0.0017	0.9994

Pharmacokinetic parameters

Pharmacokinetic parameters are derived from data by mathematical calculations[1].

The overall absorption process is considered to be a single first order process.

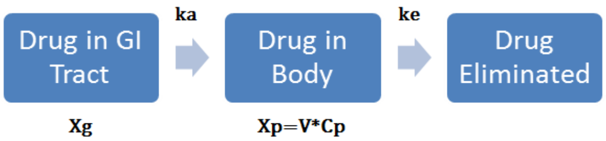

Figure X. Representing Oral Administration, One Compartment Pharmacokinetic Model

Where Xg is the amount of drug to be absorbed, Xp is the amount of drug in the body, ka is the first order absorption rate constant, ke is the elimination rate constant.

$$\frac{dXg}{dt} = -ka * Xg$$

Equation 1 Differential Equation for Amount Remaining in GI Tract

$$\frac{dXp}{dt} = ka * Xg - ke * Xp$$

Equation 2 Differential Equation for Amount of Drug in the Body

The integrated equation for Cp versus time is shown in Equation 3.

$$(Cp)t = \frac{F * Dose * ka}{V * (ka - ke)} * \left(e^{-ke*t} - e^{-ka*t}\right)$$

Equation 3 Drug Concentration after an Oral Dose

Where F is the fraction of the dose absorbed, V is the apparent volume of distribution

$\frac{F*Dose*ka}{V*(ka-ke)}$ is the intercept of the plasma drug concentration versus time plot and its slope is $\frac{-ke}{2.303}$. The terminal slope is then back-extrapolated to the concentration axis. By means of the method of residuals we subtract the observed concentrations on the upswing of the curve (called absorption phase) from the extrapolated line.

Applying the method of residuals, the plot of the difference between extrapolated and observed plasma concentrations against time, on semilogarithmic, should yield a straight line which allows determination of the first-order absorption rate constant, using the equation

ka=-slope*2.303

[1] Mohsen A. Hedaya. *Basic Pharmacokinetics* 2nd edition. 2012. Chapter 6 Extravascular routes of drug administration, pp. 105-126

Equation 4 Absorption rate constant

The equation for the time of peak can be derived by setting the rate of change of Cp versus time, dCp/dt, to zero:

$$t_{max} = \frac{1}{(ka - ke)} * \ln\left(\frac{ka}{ke}\right)$$

Equation 5 Time of Peak Concentration after an oral dose

The peak plasma concentration $(Cp)_{max}$ occurs when time is equal to t_{max}.

Area under the concentration-time curve from time zero to time t ($AUC_{0\text{-}t}$), where t is the last measurable time, was calculated by trapezoidal rule:

Area under the concentration-time curve from time zero to time infinity ($AUC_{0\text{-}\infty}$) was calculated with the formula:

$$AUC_{0\text{-}\infty} = AUC_{0\text{-}t} + \frac{Ct}{ke}$$

Where Ct is the last measurable drug concentration.
